# Interfacing mechanistic and breeding scheme simulation to predict selection response on lactation efficiency in dairy cows under different nutritional environments

**DOI:** 10.1186/s12711-025-01013-w

**Published:** 2025-11-13

**Authors:** A. Bouquet, M. Slagboom, J. R. Thomasen, M. Kargo, N. C. Friggens, L. Puillet

**Affiliations:** 1 QGG Center, Aarhus University, C.F. Møllers Allé 3, 8000 Aarhus C, Denmark; 2 Viking Genetics, Ebeltoftvej 16, 8960 Randers SØ, Denmark; 3 INRAE, AgroParisTech, Université Paris-Saclay, UMR MoSAR, 91120 Palaiseau, France; 4 PEGASE, INRAE, Institut Agro, 35590 Saint-Gilles, France

## Abstract

**Background:**

Predicting selection response for lactation efficiency in dairy cows is challenging, as the expression of this complex trait depends on dynamic interactions between the ability of cows to acquire nutrients and allocate them to different life functions. Moreover, the relative emphasis of these components may change due to energetic trade-offs between life functions when kept in limiting environments. The objective of this study is to present a new approach combining mechanistic and breeding scheme simulations to predict selection response on components of lactation efficiency of dairy cows under a non-limiting nutritional environment and when transferred to a limiting environment with a moderate feed restriction. These predictions were compared to the ones obtained with the conventional method used in quantitative genetics considering a typical dairy cattle breeding scheme and several breeding goals (BG) aiming at improving milk production, lactation efficiency and fertility.

**Results:**

In the non-limiting environment, selection responses predicted by the two methods differed for both milk production and fertility. The sign and magnitude of differences depended on BGs. Selection response predictions were consistent only for BGs that did not change much the body reserve mobilization patterns of cows, and hence their conception probability. Indeed, pregnancy status impacted energy allocation of cows and consequently milk production, more energy being allocated to lactation in case of reproductive failure. Differences in selection response between modelling approaches were slightly increased when cows were reared in the limiting environment. Overall, the choice of prediction method led to substantial BG reranking with respect to selection response on milk production and fertility. Mechanistic-based predictions of selection response for lifetime efficiency were also sensitive to the nutritional environment and BG.

**Conclusions:**

Combining mechanistic and genetic modelling is a promising approach to benchmark breeding strategies of dairy cow lactation efficiency and better anticipate the impact of changes in energetic trade-offs induced both by selection and changes in the nutritional environment. Moreover, the simulations of phenotypic trajectories over cow lifetime before and after selection enabled a better understanding of the mechanisms underlying genetic improvement.

**Supplementary Information:**

The online version contains supplementary material available at 10.1186/s12711-025-01013-w.

## Background

Large gains in milk production have been achieved by the dairy industry over the past decades due to synergistic advancements in genetic selection, nutrition, and management practices [1]. Selective breeding has been particularly successful in improving the performance of dairy cattle populations in controlled and favourable environments. However, the sustainability of such a selection strategy is questioned because it relies on the availability of high-input nutritional environments. Concerns have been raised about the possibility to indefinitely improve nutritional environments provided in intensive production systems to sustain high production levels [1]. The advent of climate change is also expected to increase the frequency and intensity of climatic events [2], making dairy production systems more vulnerable, especially those relying on grass production [3]. Other concerns pertain to the ability of specialized dairy breeds to adapt to more challenging and diversified production systems in future. Indeed, high-yielding cows are expected to be more sensitive to energy restrictions due to exacerbated energetic trade-offs. Trade-offs are linked to the allocation of energy intake to biological functions of the cow under feed restriction. Increasing allocation to one function is at the expense of another one. In the case of energy restrictions, energy is then allocated in priority to certain biological functions. Energy allocation priorities are genetically driven with variability existing both within and between breeds [4]. Intense selection on milk production over the past decades is expected to have favoured cows with higher energy allocation to lactation at the expense of fitness functions. This could partly explain the undesirable trends observed on reproduction, health and resilience traits in specialized dairy breeds, especially when breeds selected in intensive production systems are reared in more constrained ones [5]. Indeed, if feeding practices do not align in breeding and production herds, energetic trade-offs of different magnitude may occur in both environments. The best cows in the breeding environment may not be the best ones in the production environment because they allocate too much energy to productive vs. fitness functions, compromising then their productive lifespan. To define breeding programs that are sustainable in the long run, it is critical to get tools to predict selection response whilst anticipating any change in energetic trade-offs that could generate such genotype-by-environment (GxE) interactions.

The standard methodology to predict selection response in animal breeding complies with the assumptions of the genetic infinitesimal model. This approach is largely statistical by nature and does not consider any biological information (e.g. energetic trade-offs) to model genetic and phenotypic variances of traits [6]. Nonetheless, the set of genetic parameters estimated for the traits of interest integrates the magnitude of nutritional constraints that influences trait (co)variances. Selection response predictions are therefore valid for the environment in which genetic parameters were estimated considering that energetic trade-offs occurring at present remain stable over time. In the case of known GxE interactions, genetic gain transferred from breeding to production farms is generally predicted as a correlated selection response considering traits recorded in each environment as different but genetically correlated. This assumption can hold for short-term predictions but is likely to be violated in the longer term. Indeed, genetic correlations estimated between environments at a given time may not be able to reflect how trade-offs evolve in future within each environment.

Interfacing mechanistic and genetic models is a promising approach to predict selection response whilst alleviating some of the limitations previously described [7]. Mechanistic models have been developed to predict the adaptive response of dairy cows to changes in the nutritional environment by explicitly modelling the biological processes underlying lactation efficiency [4]. This hierarchical network of traits can be used to describe how genetically-driven elementary traits and environmental factors interact to shape the mean and variance of complex traits [7]. The adequate integration of genetic effects in mechanistic models has been a major obstacle to simulate inter-individual variability both within and across environments [4]. Indeed, it requires first to identify the genetic drivers of phenotypic trajectories for the traits under investigation and then to calibrate the model to simulate realistic trajectories over the lifetime of individuals and for a range of environmental conditions [8]. Recently, the AQAL mechanistic bioenergetic model of a dairy cow has been developed and integrated in a tool that can simulate phenotypic trajectories for a population of cows under contrasted nutritional environments [9, 10]. Simulated phenotypes comprise energy-corrected milk production, feed intake, body weight, body reserves and reproduction events. This model is based on the acquisition and allocation theory [11–13]. Phenotypic responses of cows to changes in the nutritional environment depend not only on the change in feed availability but also on their innate acquisition potential and energy allocation priorities, which are under genetic control. The amount of energy allocated to life functions is dynamically determined based on the achieved energy intake and energy allocation priorities using systems of differential equations. Thus, GxE interactions can emerge as simulation outcomes resulting from changes in energy allocation caused by energetic trade-offs in the case of limiting energy intake. Such GxE interactions were shown by simulation on milk production and lifetime lactation efficiency defined as the ratio between energy allocated to milk production and total energy intake [9].

The aim of this study was to interface the AQAL model and a breeding scheme simulation tool to predict selection response on lactation efficiency in dairy cattle. Selection responses predicted with this mechanistic-based modelling (MM) approach were compared with the ones obtained with the conventional modelling approach. This comparison was carried out for a panel of breeding goals (BG) with different emphasis on milk production, lactation efficiency and fertility. As often in real situations, we considered that cows were selected within a non-limiting nutritional environment and could perform either in the same environment or in a limiting nutritional environment. We hypothesized that both prediction methods will consistently rank BGs when the nutritional environment remains non-limiting over time. In contrast, if cows selected in a non-limiting environment are reared in a limiting nutritional environment, changes in energetic trade-offs will progressively shift allocation priorities of cows. Thus, we hypothesized (1) differences in selection response between prediction methods with larger differences for BGs that generate higher energetic trade-offs, and (2) re-ranking of BGs for the traits most affected by energetic trade-offs, i.e. milk production and lifetime lactation efficiency.

## Methods

### Principles of the mechanistic model

**Fig. 1:**
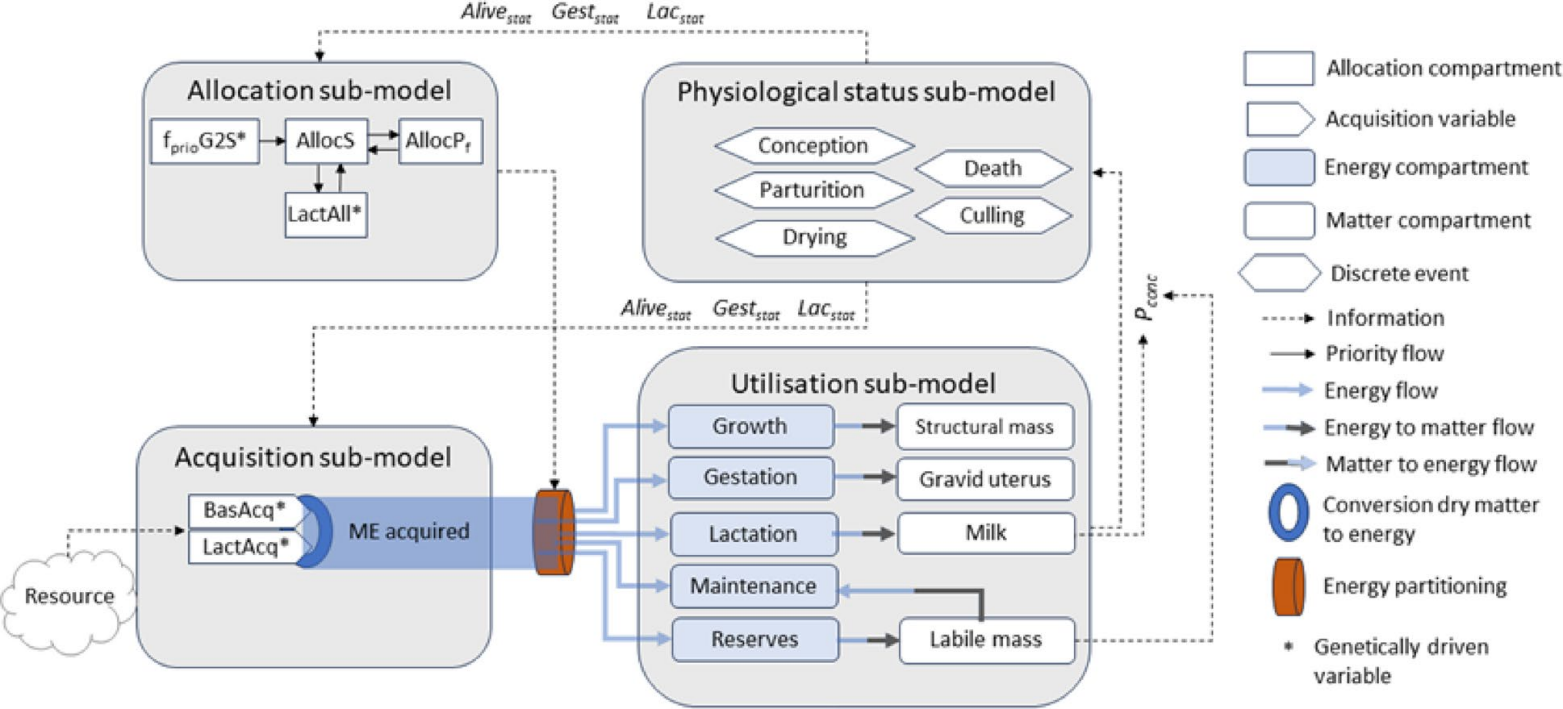
Schematic description of the conceptual bioenergetic model of a dairy cow in AQAL simulation software. Adapted from Fig. in [10]

The AQAL bioenergetic model simulates phenotypic trajectories of milk production and feed efficiency traits over the lifetime of a dairy cow depending on metabolizable energy (ME) available in the environment [10]. Flows of energy and information between the different compartments considered in the model are represented in Fig. 1. Population-wise variability of traits was induced by assuming genetic and phenotypic variance for a set of four input parameters describing energy acquisition and allocation (AA) strategies of cows. Acquisition parameters correspond to the maximal intake of a non-lactating cow at maturity (basal acquisition: BasAcq, in kg DM/d) and the increase of feed intake during lactation (lactation acquisition: LactAcq, in kg DM/d) expected, for both parameters, in a non-limiting environment. Allocation parameters correspond to the rate of transfer of energy from growth to survival (f_prio_GS, dimensionless) defining the allocation priority to structural mass growth, and the allocation to lactation (LactAll, dimensionless) defining the energy allocation to milk production at the beginning of lactation. Mean values of these four parameters were determined by a calibration procedure using real data and were set to 7.00 kg DM/d, 10.25 kg DM/d, 0.0035 and 0.56, respectively, as described in [9].

### Simulation of environments and phenotypic trajectories

The effect of the nutritional environment on the means and (co)variances of traits was modelled through stochastic simulations. Daily dry matter intake (DMI) expected in the non-limiting environment was computed as the sum of DMI related to maintenance and lactation whose trajectories were tuned by genetically-driven basal and lactation acquisition variables [10]. In limiting environments, achieved DMI could be lower than expected DMI specifically when expected DMI exceeded the daily feed DM offer. The ME content of the diet was modelled to reflect seasonal variability of nutritional resources and set according to real data collected on a research farm in New Zealand [14]. This input parameter was the same across scenarios. It varied from 10.85 to 12.45 MJ / kg of DM with a yearly average of 11.70 MJ / kg of DM. Scenarios with different nutritional constraints were simulated by varying the DM offer. In the non-limiting nutritional environment, called “high & stable” (HS) scenario in [9], cows had ad libitum access to feed. An alternative nutritional environment was considered so that DM offer was reflecting seasonal variations of resource availability and may therefore be limiting for high acquisition cows. It corresponded to the “moderate & stable” (MS) scenario in [9]. The yearly average DM offer was 12.2 kg DM / d per cow with values ranging from 10 to 16.8 kg DM / d depending on seasonality of grass production. A detailed comparison of cow phenotypes simulated in both nutritional environments can be found in [15]. Cows were reared as a single cohort considering a production system with seasonal calving as in [9], the mating season lasting for 10 weeks. Gestation lasted 282 days, and cows were dried off 90 days before calving or at latest 8 weeks before the start of calving season. The first mating of heifers occurred at 424 days of age. Cows were culled if they were not pregnant during the mating season or if they depleted their body reserves.

Energy allocated to each life function (lactation, growth, maintenance and body reserves) was simulated over cows’ lifetime using systems of differential equations following the principles of mass action laws [10]. It depended on the net energy acquired by the cow, defined at each timestep by the cow capacity to acquire resources (BasAcq and LacAcq) and the DM offer, and life function-specific allocation coefficients whose time trends were tuned using LactAll and f_prio_GS allocation priorities. Energy allocation to gestation was also included but no inter-individual variability was assumed around the trajectory of this allocation coefficient. Further details about energy allocation in the AQAL model can be found in [10].

Population-wise variability around the mean phenotypic trajectories resulted from different combinations of phenotypic values sampled for the genetically driven AA input traits and achieved energy intake as described in [9, 10]. First, true breeding values (TBVs) were sampled for the four AA input traits for each cow in the population given a simple pedigree structure as described in [9]. The population comprised 20,000 cows structured in 200 half-sib families. They were obtained from 200 unrelated sires having each 100 daughters with unrelated dams. Phenotypes for AA input traits were then reconstructed by adding a residual term to TBVs. We assumed a heritability value of 0.35 and a phenotypic CV of 0.10 for all AA input traits, and no genetic nor residual correlation between them [9]. These parameters were chosen to simulate datasets of performances with realistic genetic parameters based on a sensitivity analysis [15].

### Mechanistic-based approach to predict selection response

Trajectories for complex traits such as milk production, feed efficiency or body reserves were modelled at the phenotypic level and were the results of a given combination of AA input trait values and a nutritional scenario. Therefore, no TBVs were calculated for complex traits in AQAL, preventing us from directly predicting genetic gain from stochastic simulations. Instead, we assumed that selection on complex traits induced a correlated selection response on AA traits. This correlated selection response was predicted assuming the standard infinitesimal model and genetic parameters estimated using phenotypes and pedigrees in [16]. Updating the mean genetic values and variances of AA input traits in AQAL with the predicted correlated responses enabled the simulation of selection effects on cow complex phenotypes.

Our MM-based method comprised three steps to predict selection response. First, phenotypic performances were simulated with AQAL for a pedigreed population of cows considering the above-mentioned set of AA parameters and the two nutritional environments. Simulated data were used to estimate genetic parameters between AA input traits and complex traits simulated in the two nutritional environment [16]. Second, the ADAM breeding scheme simulation software [17] was used to predict the correlated selection response expected on AA traits for each BG in the non-limiting nutritional environment. We considered a typical dairy cattle breeding scheme structure (described below) and genetic parameters estimated using phenotypes simulated with the mechanistic model. Finally, phenotypic trajectories expected after 10, 20 and 30 years of selection (i.e. about 4, 8 and 12 generations) were simulated by updating means and variances of AQAL AA input traits with expected correlated selection responses based on ADAM simulations. The selection response for each individual trait was derived as the change in phenotypic performance before and after selection assuming no change in the feed offer pattern in each nutritional environment. Selection response transferred to the limiting nutritional environment was estimated as the change in phenotypic performances simulated with initial and updated AA input traits and considering DM offer of the limiting environment.

### Considered traits and estimation of genetic parameters

Complex phenotypes were derived from trajectories of the 20,000 simulated cows. Simulated phenotypes comprised BW at first calving (BWcalv1, in kg) and four traits measured in third lactation: energy corrected milk production (Milk, in kg), average daily dry matter intake (DMI, in kg/d), lactation efficiency (Lact_Eff, in %), expressed as the ratio of energy allocated to milk production over total energy intake, and interval from first insemination to conception as a fertility trait (IFC, in days). IFC was the result of a stochastic process based on the probability of conception that was estimated based on body reserves, energy balance and milk production at the time of mating [10]. To make milk production phenotypes comparable between selection scenarios, cumulative milk production was estimated for 280 d (average lactation duration). For shorter lactations, a cubic smoothing spline with 8 knots was fitted to individual trajectories, and milk production was predicted for days without records using the predict.smooth.spline function in R [18]. Phenotypes were only considered for lactations longer than 220d.

Genetic parameters were estimated with ReML between all AA input traits and output traits simulated in the two environments. Effects in the linear mixed models were presented in detail in [16]. Estimated genetic correlations were close to 1 between BasAcq and BWcalv1, and between LactAll and Lact_Eff as well. Hence, we considered these pairs of traits as the same traits. Finally, due to high correlations between a few traits, the genetic correlation matrix was bent to make it positive definite using the approach of [19]. Heritabilities and genetic correlations estimated between input and simulated traits are presented in Table 1.

**Table 1:** Heritabilities (on the diagonal), genetic correlations (upper triangular) and residual correlations (lower triangular) between AQAL input traits^1^ and simulated traits in the high (HS) and moderate (MS) nutritional environments

Environment	Variable^2^	AQAL input traits				HS			MS		
		BasAcq BWcalv1	LactAcq	f_prio_GS	LactAll Lact_Eff	Milk	DMI	IFC	Milk	DMI	IFC
AQAL input parameters	BasAcq / BWcalv1	***0.35***	*0.00*	*0.00*	*0.00*	0.4	0.57	− 0.09	0.12	0.64	− 0.02
	LactAcq	*0.00*	***0.35***	*0.00*	*0.00*	0.43	0.58	− 0.11	0.09	0.49	0.01
	f_prio_GS	*0.00*	*0.00*	***0.35***	*0.00*	0.05	− 0.06	− 0.44	0.20	-0.02	− 0.49
	LactAll / Lact_Eff	*0.00*	*0.00*	*0.00*	***0.35***	0.6	0.01	0.54	0.79	− 0.02	0.56
Non-limiting (HS)	Milk	0.45	0.48	0.06	0.71	**0.33**	0.51	0.31	0.67	0.57	0.33
	DMI	0.66	0.66	− 0.03	0.05	0.65	**0.34**	− 0.09	0.13	0.79	0.04
	IFC	− 0.01	− 0.02	− 0.1	0.09	0.11	− 0.05	**0.01**	0.63	0.00	0.68
Limiting (MS)	Milk	0.15	0.16	0.17	0.91	0.78	0.24	0.03	**0.29**	0.15	0.52
	DMI	0.59	0.44	− 0.01	0.02	0.53	0.61	− 0.05	0.24	**0.26**	0.00
	IFC	0.02	0.00	− 0.13	0.13	0.08	− 0.02	0.02	0.24	− 0.08	**0.01**

^1^ Heritability (diagonal elements in bold) and correlations between AQAL input traits (in italics) were defined under the assumption that the nutritional environment was in any way constraining expression of these traits

^2^ BasAcq: Basal acquisition; LactAcq: Lactation acquisition; f_prio_GS: Allocation priority coefficient to growth; LactAll: Allocation coefficient to lactation; BWcalv1: Body weight at first calving; Milk: milk production in third lactation; DMI: Daily dry matter intake; Lact_Eff: third-lactation lactation efficiency; IFC: Interval first insemination to conception

### Simulated breeding scheme

A large-scale breeding nucleus was simulated over a 30-year period with ADAM [17] to predict genetic trends expected on simulated traits and correlated responses for AA input traits. In this conventional modelling approach, TBVs and phenotypes were sampled for all traits in Table 1 assuming the infinitesimal genetic model and estimated genetic parameters.

The breeding nucleus comprised 20,000 females equally distributed in 200 herds and mated with 100 sires each year. Breeding cows had phenotypes recorded for BWcalv1, as well as Milk and IFC for each completed lactation. Breeding cows had no own feed intake and efficiency phenotypes. Accuracy of estimated breeding values was increased using genomic selection. Genomic selection was simulated using the pseudo-genomic selection approach [20], i.e. by sampling direct genomic values as a polygenic trait of high heritability (0.99) and assuming a genetic correlation between direct genomic values and TBVs equal to the accuracy of genomic breeding values (GEBV). The accuracy of GEBVs was estimated using the deterministic equation published by Goddard [21]. We considered a genome of 30 Morgans split in 30 pairs of chromosomes. Given the importance of the number of independent chromosome segments on GEBV accuracy [22], we adjusted the effective population size to 350 animals to obtain about 2000 independent chromosome segments as reported in Holstein genomic data [23]. Predictions of GEBV were assumed to be performed using a panel of 38,000 SNP markers. Moreover, we assumed a reference population of 10,000 genotyped bulls with 100 daughters each as well as 50,000 genotyped cows with performance for Milk, BWcalv1 and IFC. The reference population contained 5000 genotyped cows with DMI phenotypes. Selection index theory to combine information from bull and cow reference populations [24]. The accuracy of direct genomic values for candidates without performance was 0.68 for Milk and BWcalv1, 0.60 for DMI and 0.58 for IFC. Using sampled traits, direct genomic values and pedigree, GEBVs were predicted for all animals in the breeding scheme using DMU software [25].

Each year, the best 4000 male and 4000 female calves were assumed to be genotyped based on parental breeding values as often carried out in breeding schemes. After genotyping, the best 100 1-year old males were selected to be used as sires for one year. Within each herd, the best 100 females aged from 1 to 5 years were selected using estimated breeding values. The best 400 heifers in the population were flushed twice in a Multiple Ovulation and Embryo Transfer scheme and produced 3 offspring per mating. All matings were carried out at random. For each breeding goal, 30 replicates were simulated with ADAM. The first 20 years were considered as a burn-in period and discarded. Annual genetic gain was predicted for each trait by regressing mean TBVs of all selection candidates on their birth year. All presented results were standardized per genetic standard deviation and averaged based on 30 replicates.

### Breeding goals

All selection decisions were based on a total merit index (TMI). Breeding goals were defined to compare different selection strategies on lactation efficiency typical of specialized dairy breeds. The weights allocated to each trait in the BG are presented Table 2, together with the correlation estimated between each trait and the TMI following [26]. A baseline breeding goal (Base) was defined without any emphasis on feed efficiency. It included only milk production and IFC with weightings corresponding to the balance between production and functional traits in the Nordic TMI, i.e. aiming for a correlation of around 0.60–0.70 between milk production and TMI, and − 0.40 between IFC and TMI in the non-limiting environment [27]. In the Base scenario, TMI was moderately correlated both with BWcalv1 and DMI suggesting that selection will increase them. So, a series of BGs were defined to improve milk and fertility whilst better controlling trends on DMI and BWcalv1. The first BG included a negative weight on BWcalv1 to improve Milk and fertility and limit the increase in body weight (LimitBW). A second BG was defined by putting a negative weight on DMI to reduce the correlation between TMI and DMI (LimitDMI). With both BGs, the correlation between Milk and TMI remained high (0.63–0.64) and the correlation between IFC and TMI moderate (~− 0.40). Then, we defined two additional BGs to improve milk and fertility whilst stabilizing DMI. In the first one, we put a negative weight on both DMI and BWcalv1 to get a close-to-zero correlation between TMI, DMI and BW (LimitBoth). In the second BG, we assigned a highly negative weight on BWcalv1 to get a weak correlation between TMI and DMI (ReduceBW). In this scenario, a slightly positive weight was considered on lactation efficiency to avoid having a too low correlation between TMI and Milk. With ReduceBW and LimitBoth, the correlation between TMI and IFC remained moderate but the correlation between TMI and Milk dropped to lower values (Table 2). Finally, an extreme BG was defined by selecting only for lactation efficiency–instead of milk production–and fertility giving them equal weights (EFF).

**Table 2:** Weights of simulated traits in the different breeding goals and correlations between total merit index (TMI) and individual traits based on genetic correlations between simulated traits recorded in the high nutritional environment

Trait	Breeding goal weights							Correlation between simulated trait and TMI					
	Base	LimitBW	LimitDMI	LimitBoth	ReduceBW	EFF		Base	LimitBW	LimitDMI	LimitBoth	ReduceBW	EFF
BWcalv1	0	− 20	0	− 5	− 28	0		0.44	0.03	0.22	0.02	− 0.32	0.14
Milk	55	46	46	41	28	0		0.69	0.64	0.63	0.43	0.27	0.46
DMI	0	0	− 22	− 25	0	0		0.54	0.35	0.11	0.02	0.05	0.15
IFC	− 45	− 34	− 32	− 29	− 34	− 50		− 0.47	− 0.42	− 0.4	− 0.37	− 0.41	− 0.41
Lact_Eff	0	0	0	0	10	50		0.14	0.22	0.26	0.4	0.25	0.41

^1^ BWcalv1: Body weight at first calving; Milk: milk production in third lactation; DMI: Daily dry matter intake; Lact_Eff: third-lactation lactation efficiency; IFC: Interval first insemination to conception

### MM-based predictions of selection response

In the MM-based approach, selection response was estimated for each complex trait as the mean change in phenotypic performance within a given nutritional environment by re-simulating phenotypes with the AQAL mechanistic model observed after 10, 20 and 30 years of selection. Selection response was converted to annual responses and standardized per genetic standard deviation to be comparable with conventional estimates. To reduce computation time, we re-simulated populations of 2000 cows with updated AA input values for each time horizon (10, 20, 30 years). The mean AA input values were calculated by adding the predicted correlated selection response to the initial set of mean values determined by calibration. The heritability of AQAL input trait i (h_i_^2^^′^) was also updated to account for the Bulmer effect as $\:{h}_{i}^{2{\prime\:}}=\frac{{\alpha\:}_{i}}{{\alpha\:}_{i}\:+\:\frac{1-{h}_{i}^{2}}{{h}_{i}^{2}}}$ where α_i_ is the reduction factor in genetic variance for AA trait i that is due to the Bulmer effect so that σ_g_^2^^′^ = α_i_.σ_g_^2^. This reduction factor was estimated considering the average genetic variance observed in the cow population after discarding the burn-in period in ADAM stochastic simulations. In this calculation, we assumed that selection did not change residual variances of traits to update phenotypic variances. Changes in genetic correlations between AA input traits induced by selection were small (≤ 0.04 in absolute value) and were hence ignored.

To help interpret results and get a better understanding of the consequences of selection, average phenotypic trajectories of third-lactation cows were plotted for DMI, milk production, body weight and body reserves for each BG. Body reserves reflected the energy balance of cows over the lactation and were estimated as the ratio of labile mass to empty body weight [9]. The average number of lactations completed by all simulated cows and their lifetime efficiency was also estimated for each BG and nutritional environment. Lifetime efficiency (Life_Eff) was defined as the ratio of the total energy allocated to milk production over the total energy intake over their whole life from birth to culling. All graphs were created with ggplot2 [28] and ggpubr [29] packages in R [18].

## Results

### Selection response predictions with the conventional approach

Annual genetic gains estimated for the different BGs using the conventional breeding scheme simulations are presented in Table 3. Generation intervals were similar in all scenarios spanning from 2.4 to 2.5 years. Selection on BGs without any constraint on BWcalv1 led to high genetic gain on this trait. It was lower in other scenarios and negative for the ReduceBW BG in accordance with weights attributed to the different traits. Genetic gain predicted for Milk was high in the Base, LimitBW and LimitDMI BGs, and more limited with other BGs. Genetic trends on DMI were positive in all scenarios with the highest increase in the Base scenario. The genetic trend of DMI was close to zero only with the ReduceBW BG. Genetic gain predicted for IFC was favorable yet limited with all breeding strategies. Trends were slightly more favorable with the EFF, ReduceBW and Base BGs. Correlated selection responses expected in the MS environment following selection in the HS environment are also presented in Table 3. Irrespective of the BG, annual genetic gain on Milk was more than halved in the MS environment compared to the HS environment. The lowest selection responses on Milk were obtained with the ReduceBW and EFF breeding goals. Genetic trends on IFC were close to zero in all six scenarios.

**Table 3:** Annual genetic gain (standardized per genetic standard deviation units) predicted with the standard approach in the non-limiting (HS) breeding environment and also after being transferred to the constrained (MS) nutritional environment

Environment	Variable	Breeding goals					
		Base	LimitBW	LimitDMI	LimitBoth	ReduceBW	EFF
Non-limiting	BWcalv1	0.28	0.12	0.19	0.04	− 0.08	0.23
	Milk	0.31	0.29	0.31	0.23	0.14	0.18
	DMI	0.30	0.22	0.23	0.12	0.04	0.20
	Lact_Eff	0.16	0.14	0.17	0.11	0.07	0.24
	IFC	− 0.06	− 0.03	− 0.04	− 0.05	− 0.07	− 0.08
Limiting environment	BWcalv1	0.28	0.12	0.19	0.04	− 0.08	0.23
	Milk	0.13	0.14	0.15	0.11	0.05	0.05
	DMI	0.31	0.22	0.24	0.12	0.03	0.21
	Lact_Eff	0.16	0.14	0.17	0.11	0.07	0.24
	IFC	0.00	0.01	0.01	− 0.01	− 0.04	− 0.03

^1^ BWcalv1: Body weight at first calving; MP: milk production in third lactation; DFI: Daily dry matter intake; Lact_Eff: third-lactation lactation efficiency; IFC: Interval first insemination to conception

Correlated selection responses predicted for AA traits were consistent with the emphasis put on the different traits in the BGs (Table 4). Genetic trends expected on LactAcq, f_prio_GS and LactAll were close to each other with the Base, LimitBW and LimitDMI BGs. Selection responses for these breeding goals differed mainly for BasAcq. With the LimitBoth and ReduceBW breeding goals, low to negative genetic trends were obtained for BasAcq. Correlated selection responses on LactAll were also much more limited compared to other BGs. In contrast, these BGs displayed the highest genetic gains for f_prio_GS (Table 4). Finally, correlated responses predicted for the EFF breeding goal were different from other BGs. Indeed, high genetic gain was achieved on LactAll and BasAcq, whereas genetic gains on LactAcq and f_prio_GS were the lowest.

**Table 4:** Annual correlated selection response (standardized per genetic standard deviation units) predicted with the conventional genetic infinitesimal model for acquisition and allocation input traits and the different considered breeding goals

Variable^1^	Breeding goals					
	Base	LimitBW	LimitDMI	LimitBoth	ReduceBW	EFF
BasAcq	0.28	0.12	0.19	0.04	− 0.08	0.23
LactAcq	0.19	0.22	0.18	0.16	0.15	0.05
f_prio_GS	0.08	0.09	0.10	0.12	0.12	0.07
LactAll	0.16	0.14	0.17	0.11	0.07	0.24

^1^ BasAcq: Basal acquisition; LactAcq: Lactation acquisition; f_prio_GS: Allocation priority coefficient to growth; LactAll: Allocation coefficient to lactation

Across BGs, annual inbreeding rates remained limited ranging from 0.04 to 0.14%/yr for the EFF and ReduceBW breeding goals, respectively. This difference was mainly explained by differences in accuracy of the TMI index that was lower for the ReduceBW than EFF BG. The reduction in genetic variance of AA input traits following selection was limited with all breeding goals. Indeed, at least 0.93 of the initial genetic variance was maintained between year 21 and 30 of simulations for all AA traits (results not presented).

### Predictions with the mechanistic approach

Selection responses predicted with the MM-based approach after 20 years of selection are presented in Table 5. When improved cows were reared in the HS environment, predictions were very consistent between methods and across BGs for BWcalv1, DMI and Lact_Eff. Predictions of selection response obtained with the MM-based approach for Milk were much higher than conventional predictions for the Base and EFF breeding goals, whereas they were consistent for other BGs. Unlike the conventional approach, unfavourable (positive) selection responses were predicted for IFC with the MM-based approach for most breeding goals (Table 5). The ranking of BGs for selection response on Milk and IFC was impacted by the prediction method. For example, the LimitBW breeding goal ranked first on IFC with the MM-based approach and last with the conventional method, and the other way round for the EFF breeding goal.

**Table 5:** Annual selection response (in genetic standard deviation units) predicted for the different breeding goals with the mechanistic-based method when selection was carried out in the non-limiting environment and phenotypes expressed either in the non-limiting (HS) or constrained (MS) environment

Trait^1^	MM-based approach							Difference (MM-based–conventional predictions)					
	Breeding goal^2^							Breeding goal^2^					
	Base	LimitBW	LimitDMI	LimitBoth	ReduceBW	EFF		Base	LimitBW	LimitDMI	LimitBoth	ReduceBW	EFF
Non-limiting nutritional environment													
BWcalv1	0.26	0.11	0.18	0.02	− 0.09	0.22		− 0.02	− 0.01	− 0.01	− 0.01	− 0.01	− 0.01
Milk	0.40	0.31	0.35	0.21	0.11	0.34		0.09	0.02	0.04	− 0.02	− 0.03	0.16
DMI	0.32	0.24	0.26	0.13	0.05	0.20		0.02	0.02	0.03	0.02	0.02	− 0.05
Lact_Eff	0.17	0.15	0.19	0.13	0.09	0.23		0.01	0.01	0.02	0.02	0.01	− 0.01
IFC	0.05	0.01	0.04	0.01	− 0.02	0.15		0.11	0.04	0.08	0.06	0.05	0.23
Constrained nutritional environment													
BWcalv1	0.27	0.11	0.18	0.02	− 0.09	0.22		− 0.01	− 0.01	− 0.01	− 0.01	− 0.01	− 0.01
Milk	0.22	0.20	0.23	0.18	0.12	0.27		0.09	0.06	0.08	0.07	0.07	0.22
DMI	0.12	0.11	0.11	0.07	0.02	0.11		− 0.18	− 0.19	− 0.19	− 0.04	0.00	− 0.10
Lact_Eff	0.18	0.17	0.21	0.15	0.11	0.24		0.02	0.03	0.04	0.04	0.03	0.01
IFC	0.08	0.05	0.08	0.00	− 0.03	0.15		0.08	0.04	0.07	0.01	0.01	0.18

^1^ BWcalv1: Body weight at first calving; MP: milk production in third lactation; DMI: Daily dry matter intake; Lact_Eff: third-lactation lactation efficiency; IFC: Interval first insemination to conception

^2^ Breeding goal weights for the different traits are summarized in Table 2

The increase in DMI predicted with the MM-based approach in the constrained environment was more limited than expected with the conventional approach. Indeed, in that nutritional environment, feed intake of cows could not be increased during periods where it was already constrained (Table 5). Even though differences in selection response are substantial when expressed per genetic standard deviation, they corresponded to small phenotypic variations due to the low genetic variation estimated for DMI under constrained energy offer. Selection responses predicted for Milk in the MS environment with the MM-approach after 20 years of selection were in general higher than with the conventional methodology. Differences in prediction were the largest for the Base and EFF BGs.

### Effect of the considered time horizon to carry out predictions

In case of linear selection response, annual predictions obtained with the MM-based approach are expected not to be influenced by the time horizon considered. This was the case for Milk, DMI and Lact_Eff in the non-limiting nutritional environment (see Additional File 1 Fig. S1a). In contrast, trends on IFC tended to be slightly more unfavorable when the considered time horizon increased, especially with the Base, LimitDMI and EFF BGs. With increasing nutritional constraint, annual selection response on DMI and Milk clearly depended on the time horizon considered (see Additional File 1 Fig. S1b). This trend suggested that annual selection response on Milk slowed down over time as the result of the constrained energy offer.

### Effect of selection on phenotypic trajectories

**Fig. 2:**
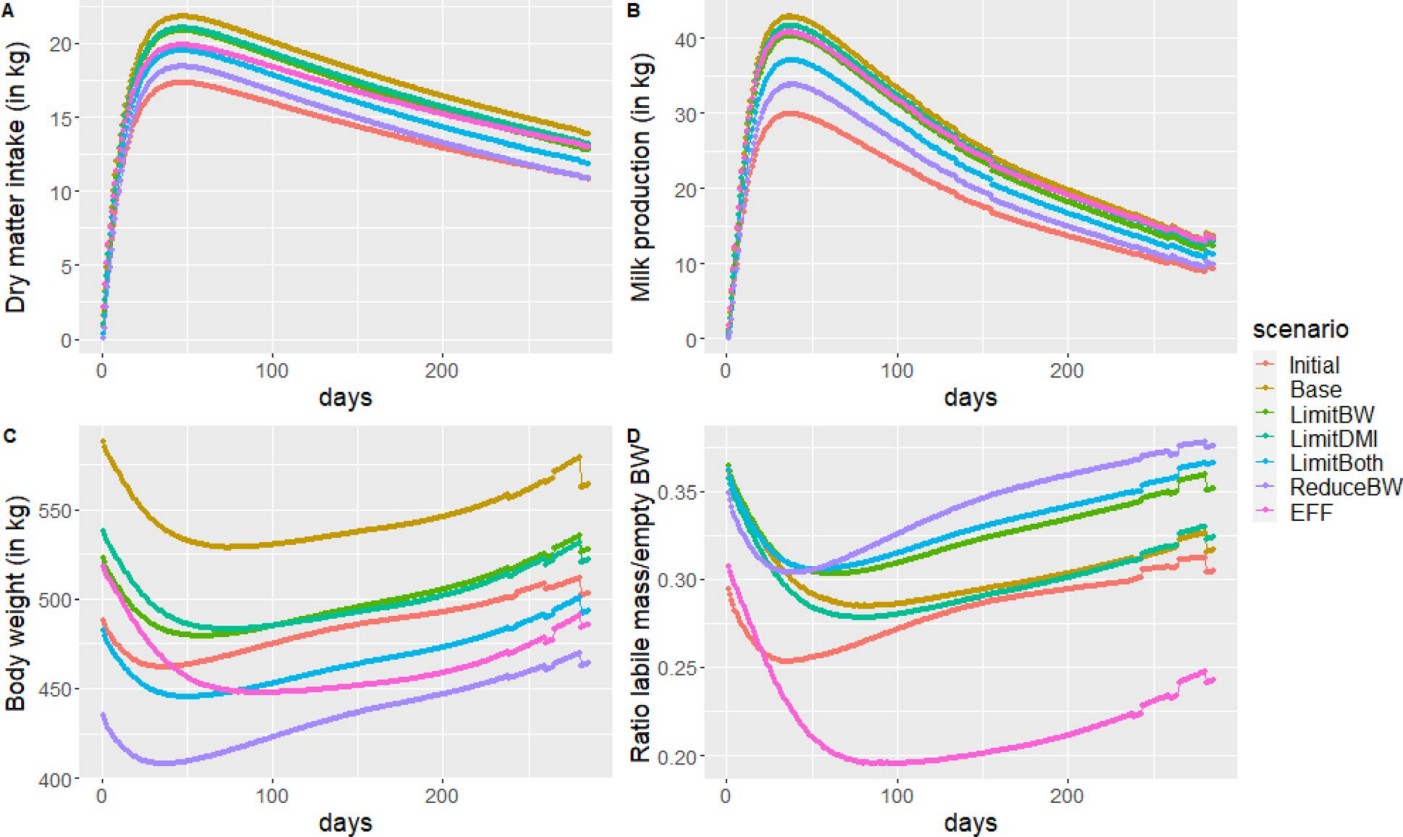
Phenotypic trajectories of third lactation cows reared in the non-limiting environment before (Initial) and after 20 years of selection for the six breeding goals. **a** Dry matter intake, **b** Milk production, **c** Body weight, **d** Body reserves

Twenty years of selection had a notable influence on the phenotypic time-trends of third-lactation cows reared in the non-limiting environment (Fig. 2). The largest increases in DMI and milk production were observed for the Base BG (Fig. 2a and b). Irrespective of BGs, increase in milk production was the highest around lactation peak. Except for the EFF BG, there is no clear difference of selection on lactation persistency. This is partly due to the fact that no genetic variation was considered on parameters controlling the persistency of lactation allocation over the lactation. The evolution of cow BW at third calving was also consistent with the constraint put on BWcalv1 in BGs (Fig. 2c). Body weight of cows selected with the EFF BG had a different time-trend during lactation with a much larger drop during the first 100 days of lactation. Time-trends of body reserve levels were also affected by BGs (Fig. 2d). Compared to the situation before selection, body reserve levels at third calving were maintained or increased in all scenarios. The dip in body reserve levels in early lactation was more pronounced and lasted longer in the EFF, Base and LimDMI breeding goals. Cows selected with LimitBW and LimitBoth BGs displayed higher body reserve levels at calving and were able to reconstitute body reserves by the end of third lactation.

**Fig. 3:**
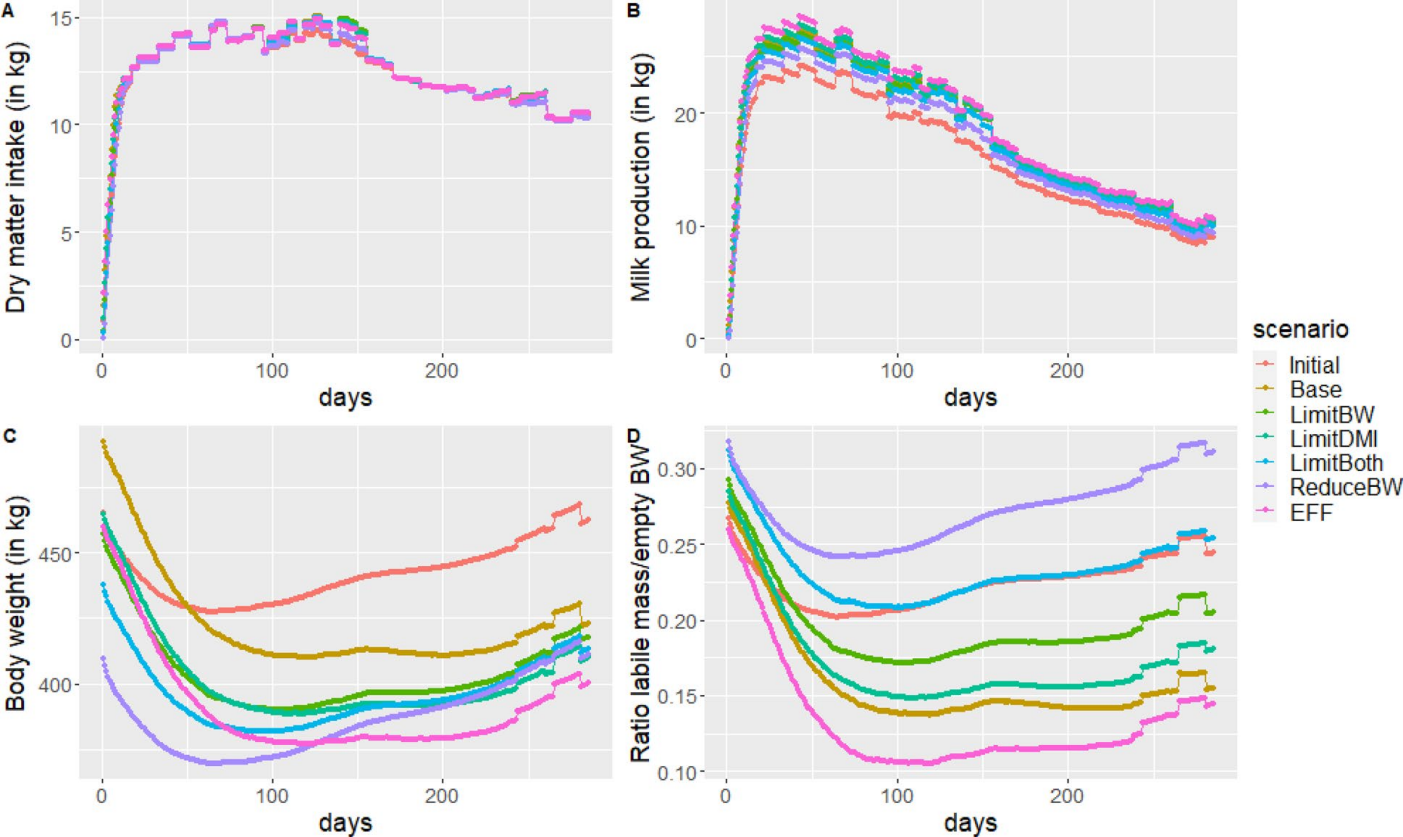
Phenotypic trajectories of third lactation cows reared in the limiting environment before (Initial) and after 20 years of selection for the six breeding goals. **a** Dry matter intake, ** b** Milk production, ** c** Body weight, ** d** Body reserves

Due to restricted feed offer in the limiting environment, DMI of third lactation cows selected in the non-limiting environment could not be increased, except for short periods of time mainly between 100 and 160d post calving (Fig. 3a). Differences in milk production trajectories between BGs were much reduced in this nutritional environment (Fig. 3b). The BW of cows at third calving was much lower than in the non-limiting environment and increased only with the Base breeding goal (Fig. 3c). The time-trend of BW during third lactation was modified compared to the situation where cows had non-limiting feed offer. Finally, the time-trend of body reserves was also much changed (Fig. 3d) with a massive mobilization in early lactation. Only cows selected with the ReduceBW were able to reconstitute body reserve levels at the end of third lactation.

### Impact of selection on lifetime efficiency

The number of lactations completed by all simulated cows and their average lifetime lactation efficiency were estimated before selection and after 20 years of selection for each BG and nutritional environment (Table 6). Lifetime efficiency was increased for all BGs in the non-limiting environment. With the EFF BG, the average number of completed lactations was strongly reduced. After 20 years of selection, Life_Eff started decreasing. Indeed, the high gains in lactation efficiency were completely offset by reduced productive life. The ranking of BGs with respect to Life_Eff depended on the number of years of selection considered, although differences in selection response remained limited. After 10 years of selection, the Base and LimitDMI BGs generated the highest selection responses that slowed down afterwards due to reduced fitness of cows. After 30 years of selection the highest lifetime efficiency was obtained with the LimitBW BG.

**Table 6:** Average number of completed lactations and lifetime efficiency of all simulated cows in non-limiting and constrained nutritional environments before selection and after 20 years of selection for the different breeding goals

Nutritional environment	Variable	Before selection	Breeding goal					
			Base	LimitBW	LimitDMI	LimitBoth	ReduceBW	EFF
Non-limiting	Mean number of lactations completed	5.86	5.35	5.87	5.34	6.09	6.46	3.77
	Life_Eff, %	37.6	42.50	43.32	43.25	42.96	42.44	40.44
Constrained	Mean number of lactations completed	4.97	3.27	4.07	3.52	4.75	5.68	2.56
	Life_Eff, %	35.4	34.47	37.80	36.32	38.92	40.08	33.63

**Fig. 4:**
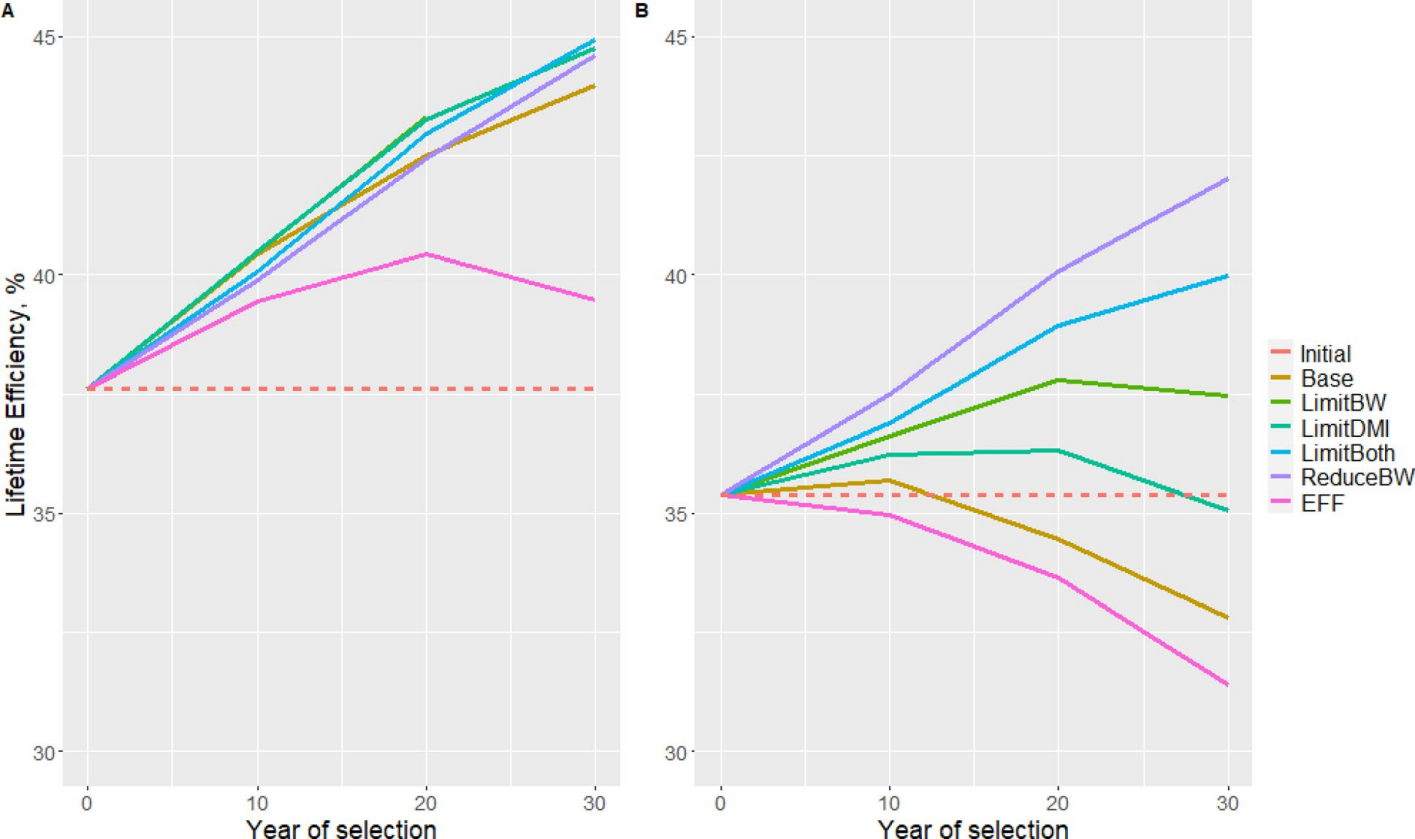
Lifetime efficiency of cows reared in the non-limiting (**a**) and limiting (**b**) nutritional environments and selected for each of the six breeding goals. Lifetime efficiency before selection is represented by the dotted line (Initial). For the six breeding goals, lifetime efficiency was predicted for cows born after 10, 20 and 30 years of selection. The predicted changes with number of years of selection depend both on change in lactation efficiency and the number of lactations completed by cows

When selected cows were reared in the constrained environment, lifetime efficiency immediately decreased in the Base and EFF scenarios (Fig. 4b) due to shorter productive lifetime that outbalanced gains in lactation efficiency (Table 6). The largest gain in Life_Eff was obtained with ReduceBW (Table 6), even though this BG achieved the lowest response on Lact_Eff (Table 5). Differences in selection response between breeding goals were larger when lifetime efficiency was expressed in the limiting environment (Fig. 4b). After 20 years of selection, lifetime efficiency went on increasing only with the ReduceBW and LimitBoth BGs though at a slower pace.

## Discussion

The creation of breeding programs maximizing nutrient efficiency and minimizing the emergence of trade-offs between production and fitness traits is highly relevant for the livestock industry. However, quantitative genetics methodology is not well equipped to evaluate how trade-offs influence selection response due to a decrease in overall fitness under current and future environments. In order to address this issue, the objectives of this study were (1) to predict selection response on lactation efficiency traits by coupling an existing mechanistic bioenergetic model of a dairy cow with a breeding scheme simulation software and (2) to compare these predictions with the ones obtained with the conventional modelling approach for a panel of BGs aiming at improving milk production, lactation efficiency and fertility under two contrasting nutritional environments.

Contrary to our initial assumption, differences in selection response were already observed between prediction methods in the non-limiting nutritional environment, i.e. in the absence of energetic trade-offs, leading to BG reranking. The magnitude of these differences depended on the effect of selection on the energetic balance of cows, as reflected by their body reserve trajectories, and consequently on their ability to reproduce. Finally, the MM-based approach revealed a high sensitivity of selection response on lifetime efficiency depending on breeding goals, the nutritional environment, and the number of years of selection. Under both nutritional environments, the highest long-term selection response was achieved with the BG having the lowest gains in third-lactation efficiency. This finding emphasizes the need for an improved quantification of energetic trade-offs and their impact on lifetime lactation efficiency in models used for the definition of balanced breeding goals in dairy cattle.

### Novelty of the mechanistic-based approach to predict selection response

In this study, we used a mechanistic model to simulate phenotypic trajectories from birth to culling of a cow population submitted to selection considering different breeding goals. The effect of selection was modelled as a correlated response on acquisition and allocation input traits that are genetic drivers of lifetime lactation efficiency in dairy cows. The mechanistic model was used as a hierarchical infinitesimal gene-to-phenotype map to predict selection response on complex traits based on the change in genetic level expected for elementary AA traits and changes in the nutritional environment (see Fig. 2 in [7]). When the nutritional environment fluctuates and becomes limiting, the causality network defined by the mechanistic model is used to dynamically reallocate energy according to individual allocation priorities. The phenotypic mean and variance of complex traits are then shaped by the cascade of intermediate mechanisms involved in lactation efficiency [9]. Some traits were modelled as the product of several intermediate traits. Genetic driven changes in the underlying components can lead to non-linearity in selection response due to trait x trait interactions [30]. For example, milk production was simulated as the product between energy intake and allocation to lactation. The overall probability of conception was also dynamically calculated for each day of lactation as the product between conception probabilities specifically due to milk production, body reserve level and body reserve mobilization [9, 31]. However, deviations from linearity observed in the present study seemed relatively limited for BGs increasing the genetic level of both acquisition and lactation allocation traits (see Additional File 1 Fig. S1). This contrasts with the conventional approach in quantitative genetics that ignores any change in (co)variances between complex traits that could arise over successive generations and be induced either by the environment or selection.

This approach makes it possible to assess how selection changes performances of a cow population over time, enabling us to detect (1) inflexions in selection response, as observed for example on lifetime efficiency, and (2) the emergence of GxE interactions. Another benefit is the possibility to explore phenotypic trajectories for all intermediate traits simulated in the mechanistic model to better understand the physiological levers underlying genetic improvement. In this study, selection responses predicted on breeding goal traits with both methods were obtained considering the same change in genetic level of AA traits, and assuming that TBVs of AA traits linearly increased over time with the MM-based approach. Thus, differences in selection response observed for complex traits between prediction methods only resulted from the non-linearity induced by the mechanistic model.

### Prediction of selection response in the non-limiting environment

We initially hypothesized that the ranking of BGs would be consistent between methods when selection was performed in the non-limiting environment and improved cows were reared in the same environment. Our rationale was that energetic trade-offs should not arise under ad libitum conditions, thereby avoiding changes in energy allocation priorities. As expected, selection responses were very consistent between methods for DMI, BWcalv1 and Lact_Eff. Indeed, these traits were highly genetically correlated with at least one of the AA input traits. Contrary to our initial assumption, substantial differences in selection response were obtained between methods for Milk and IFC. The magnitude and sign of differences depended on BGs and were clearly associated with the change in body reserve time-trends induced by selection. The largest difference was observed for the EFF breeding goal that had an extreme weight on energy allocation to lactation. Even though the environment was non-limiting, this BG exacerbated energy deficits in early lactation because the immediate increase in energy allocation to lactation could not be entirely compensated by the increase in feed intake that was slightly delayed. Both higher milk production and body reserve mobilization had a dramatic impact on conception probability due to the feedback effect in the mechanistic model. Afterwards, the pregnancy status highly affected energy allocation to lactation of cows due to the delay, or eventually absence, of energy allocation to gestation, energy that was then partly allocated to milk production. In contrast, in the conventional approach, genetic gain on milk production and fertility was predicted considering the initial set of (co)variances estimated for selected traits, i.e. ignoring causality relationships between production and fertility traits, and potential changes in genetic covariance. This example showed the interest in using the mechanistic-based approach to identify emerging properties of the system due to changes in AA priorities. Differences obtained between methods were less extreme for other BGs where the correlated responses on lactation allocation were more limited.

### Prediction of selection response in the constraining environment

When selection was performed in the non-limiting environment and the resulting cows were reared under limiting conditions, the increase in energy acquisition potential induced by selection could not be sustained by DM offer, and the realised energy intake was eventually imposed by the environment. The variance of DMI being much reduced, selection response on milk production was mostly achieved through changes in energy allocation as expected under the resource allocation framework [13]. Contrary to our assumption, differences in selection responses predicted between methods were not much increased for Milk and IFC compared to *ad libitum* conditions. Yet, we expected that feed restriction and energetic trade-offs would increase over time. The analysis of phenotypic trajectories showed that the population of simulated cows was lighter at the start of third lactation in the MS environment compared to the non-limiting conditions (Fig. 2c). Indeed, cows reaching third lactation had already experienced trade-offs during the first two lactations that diverted energy away from growing structural mass. This resulted in them being smaller, thereby lowering their maintenance requirements and limiting energetic trade-offs. Conception rates were also much lower in the MS environment than in the non-limiting environment. As mentioned earlier, having a higher number of non-gestating cows also increased milk production as energy not used for gestation was partly reallocated to lactation. Finally, the proportion of cows dropping out of simulations during the first two lactations, due to reproduction failure or depleted body reserves, was strongly increased in the limiting environment. Therefore, cows with third lactation phenotypes were not likely representative of the whole population in terms of distribution of TBVs of AA input traits.

### Assumptions and limitations of our approach

The comparison of selection response predictions was made difficult due to various assumptions made in each approach. Selection responses in the MM-based methodology could only be estimated as changes in phenotypic performance. Indeed, the AQAL model is calibrated to predict phenotypic responses over a range of nutritional environments. Sampling of TBVs is carried out only for AA input traits, defined independently of any nutritional constraint, and not for complex output traits. Hence, a drawback of our approach is that we could not partition phenotypic trends originating from genetic and environmental effects. In contrast, genetic gain was predicted in ADAM considering initial covariances for the set of traits in the BG. In the absence of any priors with respect to changes in phenotypic and genetic (co)variances over time, genetic and phenotypic selection responses are assumed to be equal. This assumption is likely to be violated if energetic trade-offs change (co)variance components over time in the breeding and target production environments. Moreover, some correlation between genetic and environmental effects might appear in the constrained environment as selection is carried out. All cows were offered the same energy amount on a given day irrespective of their acquisition or production potential. Hence, cows with the highest breeding values for feed acquisition progressively become more feed restricted than other cows, affecting thereby their energy partitioning between life functions and phenotypic selection responses [32]. Finally, as mentioned earlier, phenotypic selection responses in adult cows were partly affected by changes in the proportion of cows that were not pregnant or that dropped out of simulations due to low fitness and reproduction failure. In contrast, ignoring the effect of selection on the fitness of cows, as carried out in the conventional breeding scheme simulation, might lead to overestimation of genetic gain in particular in production systems where reproduction strongly influences herd management decisions.

In our MM-based approach, it is critical to accurately estimate correlated selection responses on AA input traits to simulate the effect of selection on phenotypic performances with the mechanistic model. In the present study, we assumed the infinitesimal model and used genetic correlations estimated between AA input and simulated output traits before the start of selection. We also considered that genetic levels for the four AA input traits evolved linearly over time. We expect this assumption to be reasonable for short-term predictions when selection is performed in a non-limiting nutritional environment. For long-term predictions or when selection is performed in a limiting environment, selection is likely to change (co)variances between input and output traits of the AQAL model. A future software development to improve the approach would be needed to regularly re-estimate variance components between traits as we perform selection to capture any deviations from linearity regarding genetic trends on AA input traits.

In this study, the mechanistic model considered genetic variation for four AA input traits that were expected to explain a large part of within-population differences in dairy cow lifetime efficiency. The mechanistic model could be enriched with extra genetically driven input traits (e.g. gestation allocation, lactation persistency) with the limitation of making it more difficult to accurately calibrate against real data [10]. More generally, as with all simulation models, it must be recognized that the results reflect to some extent the assumptions in the model architecture. In this context, the AQAL simulation tool relies on the resource allocation framework. We argue here that it provides a flexible framework to account for all energy intake and expenditures of animals during their lifetime and to integrate the main physiological constraints that ensure organisms’ stability. For instance, if genetic improvement of a trait leads to an excessive energetic cost that cannot be covered by increased intake, then energy will be diverted from other life functions with a resulting penalty on productive life [12]. It is also a framework that, by construction, can separate the biological mechanisms of resource acquisition from resource allocation. This opens up for selecting animals that would favour one or other mechanism depending on the environment in which they have to perform. Despite its convenience, there is still debate about the application of the resource allocation framework to livestock [33]. Accurate estimation of costs related to acquisition and fitness traits is also critical for modelling of energetic trade-off using dedicated experiments [34]. Moreover, genetic and phenotypic correlations between acquisition and allocation input traits may have a substantial influence on simulated traits [35, 36]. Given the absence of prior knowledge, we assumed in AQAL that AA input traits were not correlated. Further research is needed to validate the relevance of these assumptions in dairy cattle and the consequences on selection response predictions.

### Practical implications for genetic improvement of lactation efficiency

Even though the considered BGs were much simplified compared to real ones, selection responses predicted for lactation efficiency were consistent with observations in specialised dairy breeds reported in the literature. Unless strong constraints were placed on BWcalv1 and DMI to stabilize feed intake, the joint genetic improvement of milk production and fertility was supported by an increase in feed acquisition potential, an increase in lactation allocation leading to higher body reserves mobilization in early lactation, and a change in the allocation priority f_prio_G2S to favor an earlier switch of energy allocation from growth to body reserves.

The causality network described in the mechanistic model helped identify the physiological levers used for the realization of genetic gain. Similar selection responses were predicted on milk production with the Base, LimitBW and LimitDMI breeding goals with breeding scheme simulations. Yet genetic improvement was mediated by different genetic trends on the four genetically driven AA input traits with profound consequences on other traits such as body reserves. The dynamics of body reserves was critical to maintain conception probability despite the increase in milk production and body reserve mobilization in early lactation. The ability of cows to store body reserves and prepare for the next calving was then important to maintain productive life, especially in the limiting nutritional environment. In the present study, gains in lifetime efficiency were very sensitive to nutritional environment, to the breeding goal but also to the time horizon considered to make predictions. Even though increase in third lactation efficiency was positive for all breeding goals, gains in lifetime efficiency differed a lot depending on the effect of selection on cow fitness. Breeding goals with the highest selection responses on lifetime efficiency across environments were those aiming at limiting the increase in maintenance requirements rather than increasing lactation efficiency. However, this observation must be nuanced due to the high impact of fertility on lifetime efficiency in the simulated production system. Genetic improvement of this trait was somehow limited due to the low heritability estimated for IFC (0.01) that was slightly lower than values usually reported in the literature (e.g. 0.03 in Nordic genetic evaluations, NAV [37]). Indeed, the mechanistic model only accounted for the feedback effect of production and energy balance in the calculation of conception probability and ignored other polygenic factors that might influence the trait. Overall, these results emphasize the need for a better quantification of energetic trade-offs, and their impact on lifetime efficiency across the main target environments, to define dairy cattle BGs that are sustainable in the long run.

Our simulation approach provides a flexible and cost-effective way to compare long-term consequences of selection under contrasting environments. A nice feature lies in the ability of the mechanistic model to simulate phenotypes for any prospective nutritional environment, limiting thus the need for collecting data in target environments. As an example, this modelling approach was used to assess performance of dairy cows selected for resilience indicators derived from body reserves under an environment affected by more severe drought events [38]. This is highly relevant to better anticipate the consequences of current selection decisions under the lens of climate change. However, mechanistic simulations used in the present study are computationally intensive. This new approach is intended to make long-term strategic decisions and benchmark BGs or breeding schemes for prospective environments in which genetic parameters are unknown due to the absence of data. In parallel, Dekkers [39] showed how to use the equivalence between character-state and reaction norm models to predict selection responses accounting for GxE interactions along an environmental gradient. The approach of Dekkers exploits the covariance structures between multiple traits recorded in a set of discrete environments to infer genetic parameters expected along an environmental gradient within the range of measured environments. It is computationally more tractable when many scenarios need to be compared. However, the MM-based methodology can give complementary insights with respect to biological processes underlying genetic improvement and the magnitude of energetic trade-offs that may inflect selection response.

## Conclusions

This study introduced a new approach to predict selection response that combined mechanistic and genetic modelling. It permitted us to explicitly model changes in energetic trade-offs expected in the dairy cow, resulting both from selection and changes in the nutritional environment. Based on a case study, we showed that the novel and conventional prediction methods could importantly re-rank breeding goals with respect to selection response on milk production and fertility. Although the mechanistic model is still under development and breeding goals considered in this study included fewer traits than usual, results showed the interest of more holistic approaches such as this one to model the cumulated effects induced by selection and environmental changes on cow lactation efficiency over different timescales.

## Supplementary Information

Below is the link to the electronic supplementary material.

Supplementary Material 1.

## Data Availability

Phenotypic trajectories were simulated with a simulation pipeline, including the AQAL software, that was published as a data paper [15]. All simulated data are available from the corresponding author upon reasonable request.
