# Supplementary material for: Interfacing mechanistic and breeding scheme simulation to predict selection response on lactation efficiency in dairy cows under different nutritional environments
Source: Genet Sel Evol. 2025 Nov 13;57:68. doi: 10.1186/s12711-025-01013-w (PMC12616986; doi:10.1186/s12711-025-01013-w)
Supplement: Supplementary file 1 — Supplementary Material 1. [file 12711_2025_1013_MOESM1_ESM.docx]

**Supplementary Figures**


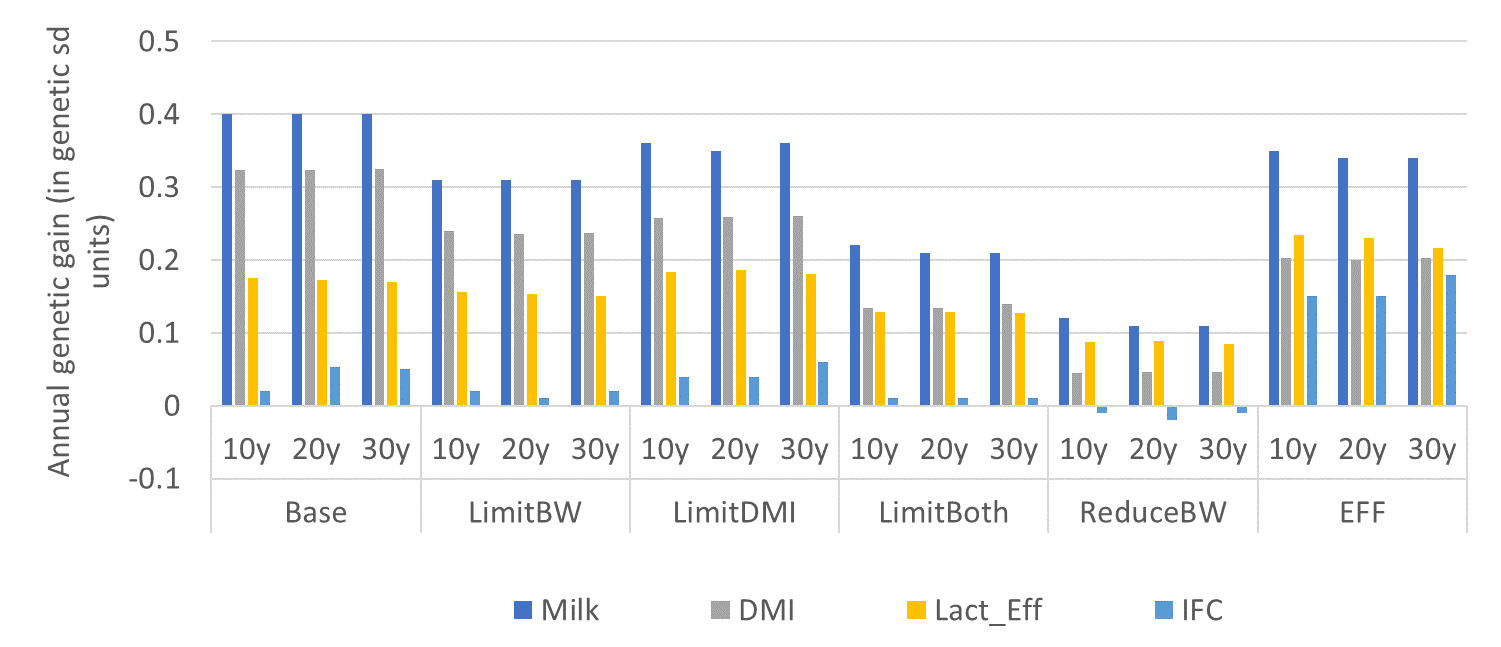


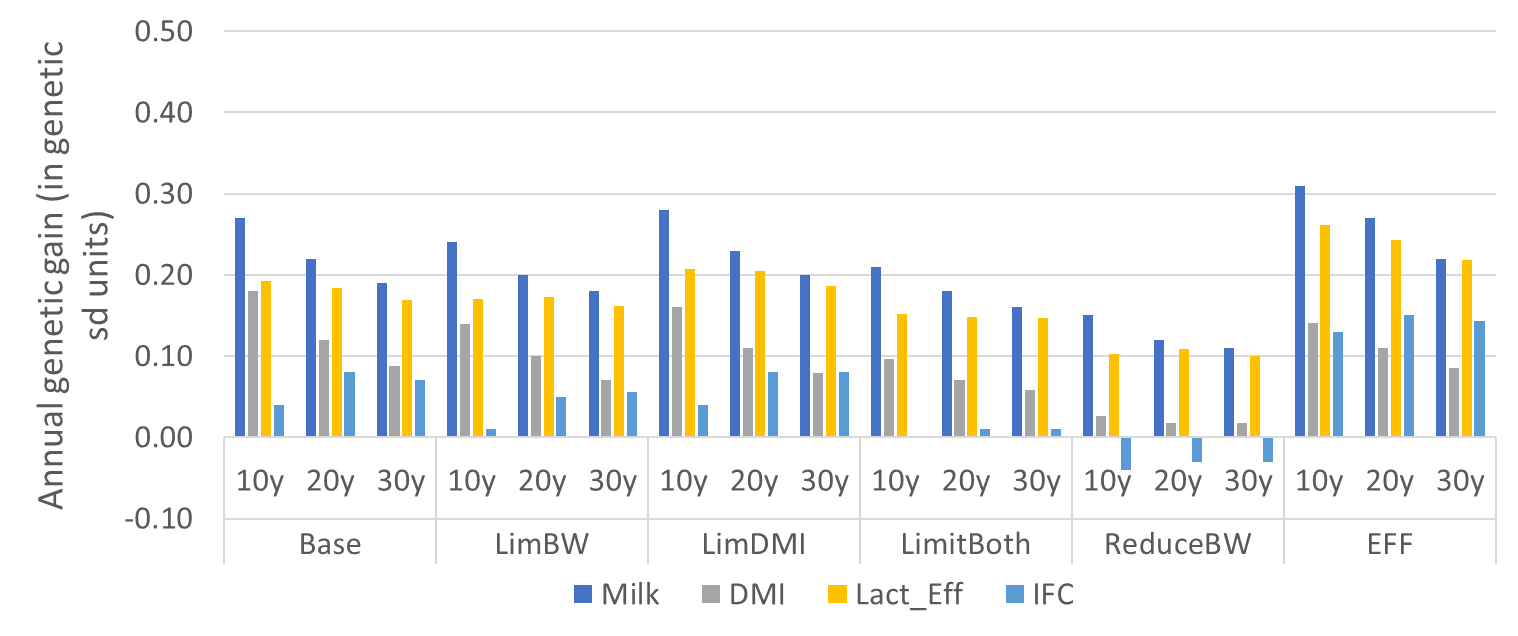


**Figure S1- Annual selection response estimated for milk production, DMI, Lactation efficiency and fertility (IFC) recorded in third lactation with the mechanistic-based approach and a time horizon of 10, 20 or 30 years in the non-limiting (top) and constrained (bottom) nutritional environments.**
